# A Qualitative Framework for Ranking the Relative Likelihood of SARS-CoV-2 Transmission Between People and Felids at Zoological Interfaces

**DOI:** 10.3390/ani16162450

**Published:** 2026-08-07

**Authors:** Ricky Tang, Jacqueline M. Norris, Carol Bency, Kimberly Vinette Herrin, Victoria J. Brookes

**Affiliations:** 1 Sydney School of Veterinary Science, The University of Sydney, Camperdown, Sydney, NSW 2006, Australia; ricky.tang@sashvets.com (R.T.); jacqui.norris@sydney.edu.au (J.M.N.); carol.bency149@hotmail.com (C.B.); 2 Small Animal Specialist Hospital (SASH), Robina, Gold Coast, QLD 4226, Australia; 3 Sydney Infectious Disease Institute, The University of Sydney, Camperdown, Sydney, NSW 2006, Australia; 4 Taronga Wildlife Hospital, Taronga Zoo, Mosman, Sydney, NSW 2088, Australia; kimberlyv@zoo.nsw.gov.au

**Keywords:** one health, SARS-CoV-2, zoonosis, risk mitigation, lions

## Abstract

SARS-CoV-2 transmission between humans and animals is an important One Health concern, particularly in settings where close human–animal interactions are difficult to avoid. We developed and applied a qualitative likelihood ranking framework for SARS-CoV-2 transmission between people and captive lions (*Panthera leo*) in an urban zoological setting. Following a structured review, scenario trees were constructed to rank direct and indirect transmission pathways using five domains: activity duration, proximity and frequency, SARS-CoV-2 survival on fomites, and environmental conditions. The framework was applied to a case study of a lion exhibit at an urban zoo in Australia. Pathway ranking depended on the combined effects of baseline exposure attributes and subsequent modifiers reflecting exposure frequency and level of natural disinfection. Although direct pathways, such as veterinary procedures, had the highest likelihood of transmission, they were differentiated by interaction duration and proximity, with frequency modification distinguishing rare, high-intensity interactions from repeated lower-intensity activities. Therefore, routine husbandry or visitor-associated exposures could increase in relative importance. Environmental modification further differentiated pathway likelihood, in particular due to solar exposure and temperature. The framework provides a transparent, practical approach for ranking transmission likelihood when data are scarce, supports consistent prioritisation of biosecurity resources, and is applicable to other emerging infectious disease threats in zoological settings.

## Simple Summary

Coronaviruses such as severe acute respiratory syndrome coronavirus 2 (SARS-CoV-2), which causes COVID-19, can spread between humans and some animal species, including animals in zoos where zookeepers, veterinarians, and visitors regularly interact with zoo animals. We developed a qualitative framework to identify and rank activities where SARS-CoV-2 could spread between humans and captive lions at an urban Australian zoo. Activities involving close and prolonged contact between staff and lions, particularly veterinary procedures and animal training sessions, had the highest likelihood of transmission. Environmental conditions, such as temperature, sunlight, and humidity, influenced virus survival in different zoo areas. The framework allows practical strategies to reduce transmission likelihood to be systematically allocated, including improved hygiene practices, physical barriers, ventilation, vaccination, and use of protective equipment. This framework will help zoos better protect animals and staff and can be adapted for use with other animal species and infectious diseases that affect humans and animals.

## 1. Introduction

SARS-CoV-2 is a member of the Betacoronavirus genus and characterised as an enveloped positive-sense RNA virus [1]. Its emergence caused the coronavirus disease 2019 (COVID-19) pandemic with >779 million cases worldwide (as of 21 June 2026), putatively following a spillover event [2,3]. The disease triggered devastating health, social and economic disruptions, prompting global endeavours to investigate the characteristics and transmission of SARS-CoV-2 and to develop preventive and control measures at an unprecedented speed [4].

At least 29 animal species have been reported to be susceptible to the virus, either after natural or experimental infection [2]. Most naturally infected animals acquired the virus through human–animal interactions, as well as extensive horizontal transmission in intensely farmed mink (*Neogale vison*) and wild white-tailed deer (*Odocoileus virginianus*) [5]. The initial anthropozoonotic introduction of SARS-CoV-2 to mink led to the emergence and amplification of mink-associated strains, causing extensive outbreaks on farms with high mortality rates [6]. Risk factors, such as high stocking density and unhygienic housing conditions, greatly contributed to the transmission dynamics [7]. Although robust evidence is lacking regarding animals as a fundamental reservoir for the COVID-19 pandemic, instances of spillback events from mink, white-tailed deer and lions (*Panthera leo*) to humans have been reported [6,8]. Individuals associated with mink farms, including residents, employees, and others in contact with them, have been infected with viral strains exhibiting animal sequence signatures. The bidirectional transmission between humans and mink affected at least 43 farms in the Netherlands, likely facilitated by shared personnel, feed suppliers and veterinarians between farms [6,9]. Free-ranging white-tailed deer serve as another example where evolutionary analyses have identified at least 109 independent human-to-deer introductions, including 39 instances of subsequent deer-to-deer transmission and three putative deer-to-human spillback events [10,11]. Collectively, these examples demonstrate that repeated human-to-animal introduction can permit sustained viral circulation, evolution, and potential spillback, reinforcing the importance of identifying transmission interfaces between humans and susceptible wildlife.

In March 2020, the first documented case of human-to-non-domestic felid transmission occurred when a tiger (*Panthera tigris*) displayed intermittent coughing and wheezing and tested positive for SARS-CoV-2 [12]. Subsequently, three tigers and three lions in the same zoo tested positive. Epidemiologic and genomic data suggest the non-domestic felids acquired the infection from symptomatic staff members during close (≤1.8 m) but not direct contact, including moving of animals between enclosures and exhibits, feeding, training sessions, enrichment activities, greetings and social interaction (for example, chuffing). SARS-CoV-2 RNA was also detected from the respiratory secretions and faeces of the seven animals, warranting further investigations of animals as a potential reservoir for secondary spillback events [13].

At another zoo, RT-PCR and whole-genome sequencing demonstrated that two zoo employees were infected with a SARS-CoV-2 strain identical to that of an infected lion on-site [8]. These findings, coupled with backward and forward tracing, suggested that at least one employee contracted SARS-CoV-2 infection from the lion, potentially directly via respiratory transmission (for example, roaring or coughing in the exhibit environment) [8]. Following detection of infection in the lion, enhanced infection-control measures were implemented, including mandatory N95 respirator use for employees caring for susceptible species, restriction of access to essential personnel, and additional PPE requirements for personnel entering the building. Collectively, these outbreaks demonstrate that close interactions between humans and captive lions may provide opportunities for bidirectional SARS-CoV-2 transmission.

Transmission routes of SARS-CoV-2 as a reverse zoonosis are postulated to be similar to those between humans, including inhalation of respiratory droplets and aerosolised particles, deposition of the viral respiratory fluids on mucous membranes and physical contact between mucous membranes and contaminated surfaces [14]. Most animal species that test positive for SARS-CoV-2 are known to have had close contact or proximity to infected humans. However, there is scarce published evidence describing the risk factors associated with the bidirectional zoonotic transmission between humans and animals [15]. Furthermore, existing resources on SARS-CoV-2 management in animals are generalised and outdated, drawing primarily from early pandemic literature [16]. Therefore, the identification of species- and habitat-specific risk factors of SARS-CoV-2 is necessary for the development of mitigation strategies to safeguard the health of animals and humans.

The objective of this study was to develop a qualitative likelihood ranking framework to support identification and prioritisation of SARS-CoV-2 transmission mitigation strategies between zoo felids and people. We conducted a structured literature review to identify potential transmission pathways between zoo felids and people, then constructed a qualitative likelihood ranking tool to assess relative transmission likelihood and identify high-likelihood transmission pathways. Lions from an urban zoo in Australia were used as a case study because lions appear particularly susceptible to SARS-CoV-2 infection [17,18] and are commonly maintained across zoological collections globally. Our aim was to provide a practical framework to support mitigation of SARS-CoV-2 transmission likelihood between people and zoo felids.

## 2. Materials and Methods

### 2.1. Identification of SARS-CoV-2 Transmission Pathways

A structured literature search was conducted to identify evidence for transmission pathways for SARS-CoV-2 infection in captive non-domestic felids. The search was conducted using MeSH headings and keywords: (COVID-19/(MeSH) OR COVID-19 OR SARS-CoV-2/(MeSH) OR SARS-CoV-2) AND (Felid* OR Feline*) in Medline (1946-present). Additional articles were also sourced by manual review of reference lists from eligible articles. Eligible articles were reviewed, and evidence relating to transmission pathways was summarised to inform scenario-tree construction.

Available evidence describing transmission pathways between humans and zoo felids was limited, with many investigations inferring transmission pathways rather than directly demonstrating them. However, genomic analyses in outbreak investigations demonstrated high viral sequence similarity between infected zoo staff and non-domestic felids, supporting human–felid transmission [12,17,19,20,21]. Zoo staff, rather than visitors, were implicated as likely infection sources in multiple investigations [5,12,17,19,20,21].

As direct evidence defining transmission pathways between humans and zoo felids remains limited, pathways recognised in humans and supported by available evidence in felids were incorporated into framework development. These included respiratory droplets, aerosols, mucosal exposure, and indirect transmission via contaminated surfaces [5,22,23]. Airborne transmission was further supported by evidence from domestic cats and ACE2 receptor homology studies [24,25].

Faeco–oral transmission was considered during framework development because viral RNA has been detected in faecal samples, gastrointestinal ACE2 expression has been demonstrated in felids, and experimental evidence indicates that SARS-CoV-2 can infect cell cultures and organoids [17,19,21,23,26]. However, strong epidemiological evidence remains lacking [27,28]. Successful transmission would require viable virus to be shed in faeces, persist in the environment, and subsequently reach a susceptible host through ingestion or oral exposure to contaminated faecal material, food, water, or surfaces. Such exposure was not identified as a routine human–lion interaction within the assessed setting. Given the predominance of respiratory transmission, the faecal–oral pathway was considered biologically plausible but insufficiently supported and of limited operational relevance for inclusion in the final likelihood ranking framework.

In May 2024, the primary author conducted a single-day site visit to observe enclosure design, husbandry practices, and interactions relevant to potential SARS-CoV-2 transmission pathways. Information regarding routine procedures, interaction durations, and activity frequencies was further informed by consultation with co-author KH, a senior zoological veterinarian involved in managing the lion collection, and a senior carnivore zookeeper.

### 2.2. Qualitative Transmission Likelihood Ranking Framework

#### 2.2.1. Scenario Tree

**Figure 1:**
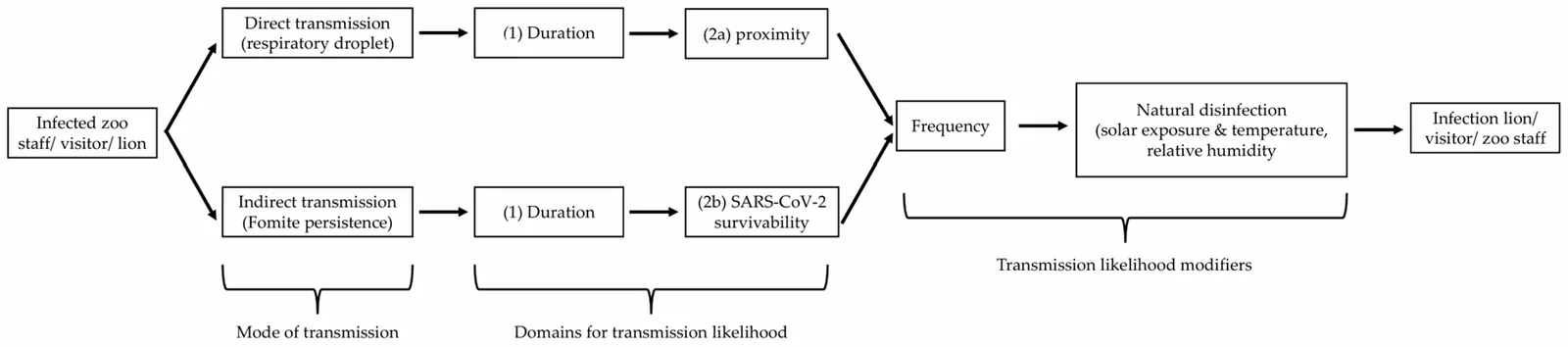
Scenario tree illustrates the domain and modifiers incorporated into the qualitative likelihood ranking of direct and indirect SARS-CoV-2 transmission pathways between people (zoo staff and visitors) and lions.

A scenario tree describing direct and indirect SARS-CoV-2 transmission pathways between people and zoo felids was developed (Figure 1). Direct transmission pathways represented exposure through direct interaction between infectious and susceptible individuals. Indirect transmission pathways represented exposure through contaminated fomites in the zoo felid environment. The framework evaluated bidirectional interaction-associated transmission opportunities rather than direction-specific transmission probabilities, because, for most activities, transmission direction did not alter the characteristics or relative ranking of the interaction. Activities considered unidirectional were identified separately.

Transmission likelihood was evaluated using a qualitative propagation framework incorporating three domains and three modifiers:

i. Interaction duration,
ii. Interaction proximity (direct pathway only),
iii. SARS-CoV-2 persistence on fomites (indirect pathway only),
iv. Frequency of interaction occurrence over a defined assessment period,
v. Solar exposure and temperature,
vi. Relative humidity.

Interaction proximity was incorporated for direct transmission pathways because closer interactions might increase opportunities for respiratory droplet, aerosol, and mucosal exposure. SARS-CoV-2 persistence on fomites was incorporated for indirect transmission pathways to account for variability in environmental survival on different materials.

Interaction frequency and level of natural disinfection were included as modifiers to account for cumulative exposure over time and environmental influences on viral persistence, respectively. Incorporation of interaction frequency allowed repeated low-intensity interactions (for example, feeding events) to be compared with infrequent but high-intensity interactions (for example, veterinary procedures) within a standardised assessment period.

Mitigation measures (for example, personal protective equipment [PPE]) were not incorporated into scenario trees or transmission likelihood estimation so that transmission pathways could initially be ranked according to inherent likelihood before considering the relative value of mitigation strategies.

#### 2.2.2. Parameterisation and Transmission Likelihood Estimation

Transmission likelihood scales for the domains of interaction duration, proximity, and SARS-CoV-2 persistence were parameterised using author-defined ordinal categories informed by established COVID-19 transmission guidance, published evidence, and biological plausibility. These categories were selected based on evidence that transmission likelihood increases with close and prolonged contact, while longer SARS-CoV-2 persistence on fomites provides greater opportunity for indirect transmission. Where the available evidence did not support precise numerical thresholds, pragmatic cut points were selected to distinguish progressively increasing exposure conditions. The selected thresholds were intended to provide a transparent and pragmatic framework for zoo-felid likelihood assessment and should not be interpreted as empirically validated cut points or estimates of absolute transmission probability [29].

The boundaries in Table 1 distinguish very brief (<30 s), brief (30 s–10 min), intermediate (>10–30 min), prolonged (>30–60 min), and very prolonged (>60 min) interactions. Similarly, the proximity categories distinguish near-contact (≤0.5 m), close-range (>0.5–2 m), intermediate-distance (>2–5 m), more distant (>5–10 m), and remote (>10 m) interactions. These boundaries provide a transparent five-level ordinal scale and do not imply that transmission likelihood changes abruptly at each cut point. The rationale for the frequency and environmental modifiers is provided in Sections Interaction Frequency Modifier and Environmental Modifiers.

The complete calculation process can be reconstructed from Table 1, Table 2 and Table 4: Table 1 assigns the initial domain categories, Table 2 combines interaction duration with proximity or fomite persistence to determine the baseline likelihood category, and Table 4 specifies the subsequent application of frequency and environmental modifiers to derive the final category. Modifiers were applied sequentially, with increases and decreases offsetting one another when acting in opposing directions; final categories were bounded by the negligible and very high categories.

Table 2 was constructed as a symmetric ordinal matrix in which the combined likelihood increased as both input categories increased. A low or negligible category in one domain constrained the combined result, while a very high baseline likelihood required one domain to be very high and the other to be at least high. This provided a consistent method for combining the two domains without implying numerical transmission probabilities.

A blank SARS-CoV-2 transmission likelihood worksheet has been provided as a Supplementary Material to demonstrate the likelihood calculation process step by step and facilitate the application of the framework by other researchers.

For direct transmission pathways, baseline per-event transmission likelihood was derived by combining interaction duration with proximity (i.e., Domain 1 × Domain 2a) between potentially infectious and susceptible individuals, using the likelihood propagation matrix in Table 2.

For indirect transmission pathways, baseline per-event transmission likelihood was derived by combining interaction duration with SARS-CoV-2 persistence on the highest-likelihood fomite encountered (Table 3) (i.e., Domain 1 × Domain 2b) using the likelihood propagation matrix in Table 2.

The modifiers, interaction frequency, solar exposure and temperature, and relative humidity were subsequently applied to account for virus persistence and cumulative exposure over time (see Sections Interaction Frequency Modifier and Environmental Modifiers and Table 4).

##### Virus Persistence on Fomites

Published evidence indicates that SARS-CoV-2 persistence varies substantially according to fomite type (Table 3) and environmental conditions (see Section Interaction Frequency Modifier). Fomite persistence estimates were used to parameterise the SARS-CoV-2 persistence domain for indirect transmission pathways. Persistence on fomites was used as a surrogate measure of relative opportunity for indirect transmission rather than a direct estimate of fomite-mediated transmission probability, recognising that successful transmission additionally depends on contamination events, survival of infectious virus, transfer efficiency, host contact, and infectious dose.

Viral persistence generally decreased with increasing temperature. For example, SARS-CoV-2 half-life on stainless steel decreased from 43.2 h at 20 °C to 12.6 h at 30 °C under 50% relative humidity conditions (Table 3).

Data describing SARS-CoV-2 persistence on hay, grass, and rock surfaces were not identified in the literature.

The highest-likelihood fomite encountered within each indirect pathway was used to conservatively and pragmatically parameterise fomite persistence in the baseline likelihood matrix, rather than summing the contributions of multiple fomites. For raw meat, temperature conditions are dynamic, encompassing frozen storage, refrigerated thawing, preparation, and subsequent placement at ambient temperature [30]. Fomite persistence was therefore classified according to the refrigerated temperature of approximately 4 °C, corresponding to a “very high” persistence rating, rather than the frozen storage temperature of −20 °C. Although the surface temperature of the meat may increase during placement and consumption, it is expected to be consumed shortly after being offered to the lions. Consequently, the meat is unlikely to remain at ambient temperature for long enough to substantially reduce viral survivability before ingestion.

**Table 3:** SARS-CoV-2 persistence estimates on fomites in zoo felid environments. RH = relative humidity; NR = not reported. * Persistence likelihood based on temperature at 4 °C.

Area	Fomite	Temperature (°C)	RH (%)	Time to Complete Decay (d)	Half-Life (h)	Persistence Likelihood Category (Table 1)	Reference
Back of house	Stainless steel	20	50	>5.96 *	43.2	High	[31]
		30	50	>1.74 *	12.6		[31]
		NR	NR	4	5.6		[32]
		22	65	7	14.7		[33]
	Wood	22	65	2	NR	High	[33]
	Concrete	21	NR	NR	7.96	Moderate	[34]
		25	NR	NR	2.54		[34]
	Raw meat	4	NR	9–21	NR	Very High *	[35,36]
		−20	NR	>20	NR		[36]
	Plastic	22	65	4–6	NR	High	[33]
		23–25	40–50	>5	NR		[37]
Public viewing area	Glass	20	50	>6.32 *	45.6	High	[31]
		30	50	>1.45 *	10.5		[31]
		22	65	4	4.8		[33]

##### Interaction Frequency Modifier

Interaction frequency modified transmission likelihood according to increasing cumulative exposure over a one-week assessment period of typical operation. Frequency modifiers allowed repeated lower-intensity interactions to be compared with less frequent higher-intensity interactions by increasing transmission likelihood along the ordinal likelihood scale.

##### Environmental Modifiers

Published evidence indicates that environmental conditions modify SARS-CoV-2 persistence, with the strongest and most consistent effects observed for temperature and solar exposure. Relative humidity was also considered, although reported effects are less consistent and appear more dependent on matrix composition and surface type.

Increasing temperature was treated as reducing viral persistence. Harapan et al. [38] demonstrated reduced SARS-CoV-2 viability following exposure to temperatures ≥40 °C, with no detectable viral viability after one hour at ≥55 °C. Similarly, Riddell, Goldie, Hill, Eagles and Drew [31] reported reduced surface persistence at elevated temperatures, with SARS-CoV-2 half-life decreasing from 1.68 to 2.74 days at 20 °C to approximately 3 h at 40 °C. Conversely, Huang et al. [39] demonstrated greater viral stability under cold conditions, with infectious virus remaining detectable after prolonged storage at −20 °C.

Solar exposure was treated as reducing viral persistence relative to baseline laboratory conditions. Ratnesar-Shumate et al. [40] demonstrated rapid viral inactivation under simulated sunlight, with substantially faster decay under ultraviolet exposure than in darkness. Reduced solar intensity was associated with slower viral inactivation.

RH was applied as a secondary environmental modifier only where values were outside the intermediate range and judged likely to enhance persistence. Smither et al. [41] demonstrated that viral decay varied according to both RH and matrix composition. Biryukov et al. [42] observed more rapid decay with increasing humidity, whereas Morris et al. [43] demonstrated a non-linear relationship between RH and viral persistence, with greatest inactivation at intermediate RH.

Environmental modifiers were applied to reflect mean monthly environmental conditions at the assessed setting, rather than the experimental conditions under which persistence estimates were originally derived (Table 4). Solar exposure categories were defined using mean daily global solar exposure (MJ/m^2^/day). Because most fomite persistence estimates used to parameterise baseline likelihood were derived under laboratory conditions without natural solar exposure, any natural solar exposure was treated as reducing persistence relative to baseline. Enclosed or indoor conditions without natural solar exposure were therefore treated as baseline rather than persistence-enhancing. Cold, refrigerated, or frozen conditions were treated as enhancing modifiers because experimental evidence indicates prolonged SARS-CoV-2 persistence under low-temperature conditions.

**Table 4:** Modifiers accounting for frequency of interaction and environmental conditions.

Modifiers	Definition	Modification
Frequency of interaction	<Once weekly	No change (0)
	Between once weekly and once daily	Increase 1 level (+1)
	>Once daily	Increase 2 levels (+2)
Solar exposure & Temperature	Strongly reducing: High mean monthly solar exposure ≥22 MJ/m^2^/day and/or mean monthly temperature ≥28 °C	Reduce 2 levels (−2)
	Reducing: Any natural solar exposure <22 MJ/m^2^/day and/or mean monthly temperature >23–<28 °C	Reduce 1 level (−1)
	Neutral: No solar exposure and mean monthly temperature 15–23 °C	No change (0)
	Enhancing: cool mean monthly temperature 10–<15 °C	Increase 1 level (+1)
	Strongly enhancing: cold conditions <10 °C, refrigerated storage, or frozen storage	Increase 2 levels (+2)
Relative humidity	RH 40–80%	No change (0)
	RH <40% or >80%, where judged persistence-enhancing	Increase 1 level (+1)

## 3. Results

### 3.1. Exhibit Environment

#### 3.1.1. Back-of-House Area (BOHA)

The BOHA is a semi-open-air space, comprised of two main areas: a housing pen complex containing six pens and a food preparation and storage area. Lions rested, underwent husbandry procedures, participated in husbandry training activities, and were fed within the housing pen area.

Zoo staff activities relevant to the transmission pathway assessment included food placement, feeding sessions, cleaning and disinfection procedures, husbandry training, veterinary assessment, and shifting animals between enclosure spaces. Food placement was considered distinct from feeding sessions. It was classified as an indirect transmission pathway because food could become contaminated during preparation and was subsequently placed within the PVA or BOHA without direct interaction or close proximity between staff and the lions. In contrast, feeding sessions were classified as a direct transmission pathway because food was delivered by a keeper in close proximity to the lion. Collectively, these activities generated opportunities for both direct and indirect transmission.

**Figure 2:**
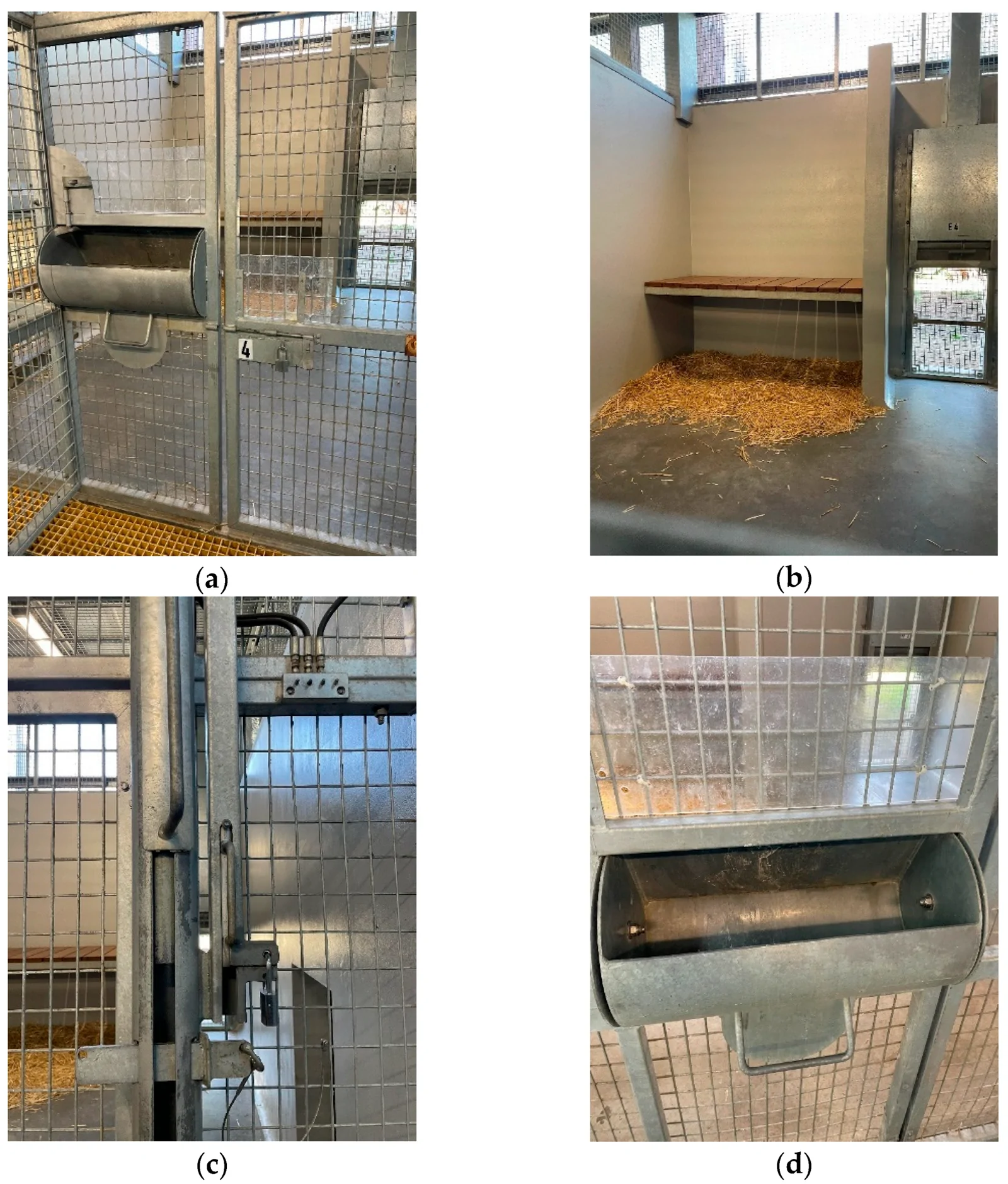
Back-of-house area in the lion habitat at the urban zoo, Sydney; (**a**) stainless steel housing pens with in-built feed trough and yellow barrier line, (**b**) an elevated wooden platform with hay for bedding, (**c**) stainless steel shifting slide on gate, (**d**) feed trough and plexiglass barrier.

Fomites identified within the BOHA included stainless steel housing gates and shifting slides, plexiglass barriers, integrated feeding troughs, elevated wooden platforms with hay bedding, concrete flooring, and raw meat associated with feeding (Figure 2a–d).

#### 3.1.2. Public Viewing Area (PVA)

The PVA comprised an open-air exhibit exposed to ambient environmental conditions. Habitat features included resting and movement areas containing wooden platforms, hay bedding, rocks, vegetation, and enrichment structures.

**Figure 3:**
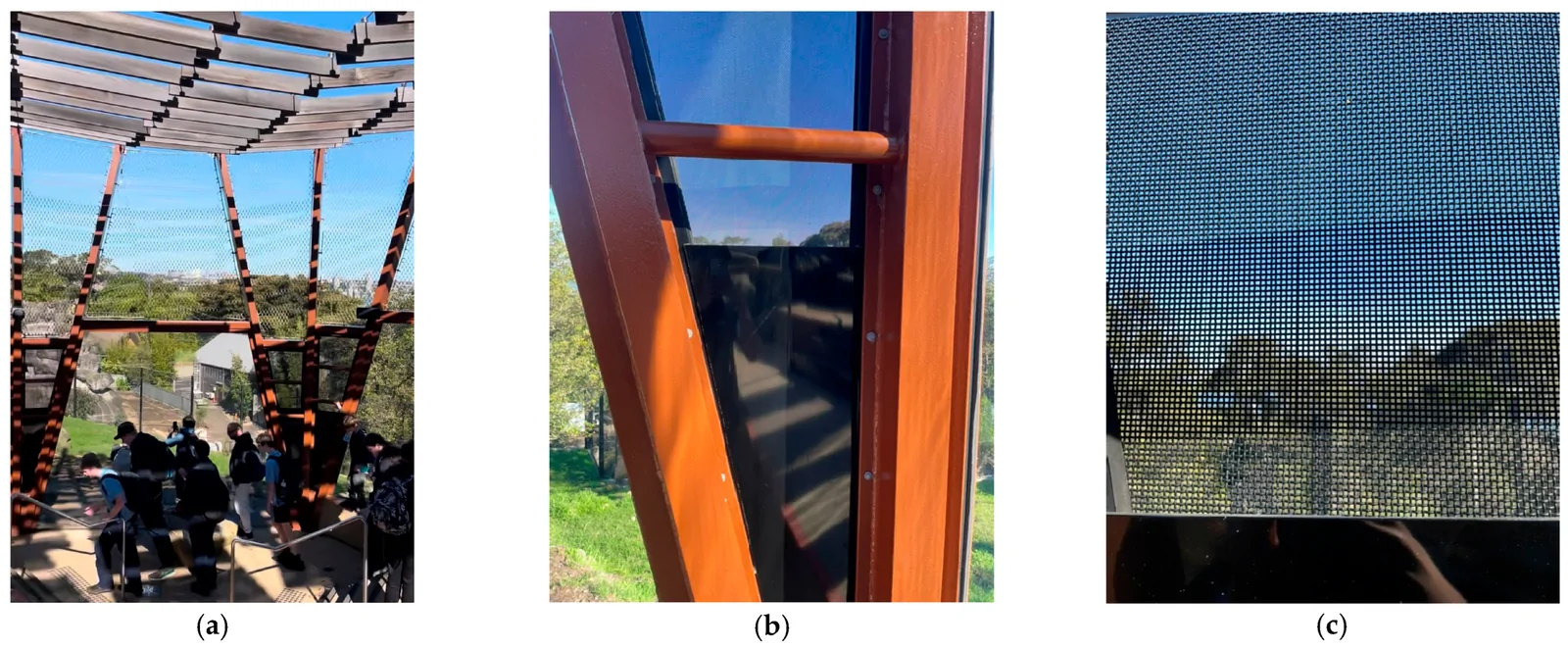
Public viewing area showing barriers in the lion habitat at the urban zoo; (**a**) glass panels, (**b**,**c**) fine metal mesh allowing shared airspace between visitors and lions.

Observed visitor-associated activities consisted primarily of visual observation adjacent to enclosure barriers. Visitors were separated from the lion enclosure by glass viewing panels and, in selected areas, fine metal mesh barriers that permitted airflow between visitor and animal environments (Figure 3).

Potential indirect transmission opportunities within visitor areas included visitor-associated surfaces and glass viewing barriers. Potential direct transmission opportunities were limited to locations where airflow between visitor and animal environments occurred.

### 3.2. Environmental Conditions

During May 2024, the mean monthly temperature was 20.6 °C (range 15.9–24.2 °C), and the mean solar exposure was 9.9 MJ/m^2^ (range 3.8–12.7 MJ/m^2^).

Throughout 2024, the highest mean monthly temperature was in January (28.3 °C; range 23.7–38.5 °C), and the highest mean monthly solar exposure was in December (26.1 MJ/m^2^; range 8.1–32.0 MJ/m^2^). The lowest mean monthly temperature was in June (17.8 °C; range 12.1–22.0 °C), and the lowest mean monthly solar exposure was also in June (8.7 MJ/m^2^; range 3.2–11.1 MJ/m^2^). The annual mean relative humidity at 9 am and 3 pm varied from 52 to 78% and from 40 to 64%, respectively. As these values remained within the predefined relative humidity category in Table 4 and did not alter the assigned environmental modifier, relative humidity was not applied separately in this case study.

The PVA was fully exposed to ambient environmental conditions. Environmental conditions within the BOHA were also partially exposed to ambient environmental conditions due to its semi-open-air, naturally ventilated design. However, these conditions were partially modified through the use of heating pads maintained at approximately 25 °C and supplementary heating lamps.

### 3.3. Likelihood Ranking

Activities at the urban zoo during May 2024, together with their associated interaction duration, proximity, fomite persistence, frequency and level of natural disinfection, were parameterised against the scenario tree and are summarised in Table 5. The qualitative likelihood propagation framework was then applied to estimate the final transmission likelihood for each activity (Table 6).

Activities that occurred in the BOHA under enclosed conditions were modified locally to approximately 20 °C through heating pads maintained at approximately 25 °C and supplementary heating lamps but were not cooled in summer.

Two activities were treated as unidirectional human-to-felid pathways and are marked with an asterisk in Table 5 and Table 6. Food placement involves staff preparing raw meat that is subsequently placed within lion enclosures without their presence; visitor-created enrichment items are handled by visitors before being placed in the lion habitat.

Among staff activities, five of six were ranked as very high likelihood in May 2024 (veterinary clinical procedures, husbandry training, feeding sessions, BOHA cleaning, and food preparation). Moving animals between enclosure spaces was ranked high. The five very high-ranked activities reached the top tier via different routes. Veterinary procedures reached very high from baseline alone, reflecting prolonged close-contact interactions during clinical work. Husbandry training reached very high from a high baseline due to its long duration and frequency. Feeding sessions, BOHA cleaning, and feed placement (to lions only) reached very high from moderate baselines amplified by cumulative exposure across multiple daily interactions.

Visitor activities ranked lower. Viewing adjacent to the mesh barrier ranked moderate, with the low baseline (brief, distant contact) partially offset by outdoor solar exposure but elevated by very high visit frequency. Enrichment item production (to lions only) ranked low, reflecting the brief, indirect, and infrequent nature of the activity.

Baseline likelihood estimates were re-evaluated under environmental conditions characteristic of January and December (high solar and temperature), and June (low solar and temperature; Table 7 and Table 8). June conditions were the same as those in May.

January and December conditions shifted every activity exposed to ambient or partially ambient conditions down by one tier: staff activities at very high dropped to high, moving animals dropped from high to moderate, and visitor viewing dropped from moderate to low. Enrichment item production, assumed to occur in a climate-controlled visitor area, was unchanged across all scenarios.

## 4. Discussion

This study developed and applied a qualitative transmission likelihood ranking framework to identify and rank plausible pathways for SARS-CoV-2 transmission between humans and captive lions in an urban zoological setting. By differentiating transmission likelihood, the framework provides a structured and practical approach for identifying high-likelihood interfaces and prioritising mitigation strategies without requiring quantitative transmission probability estimates or extensive epidemiological datasets. We identified direct staff–lion interactions as the highest-likelihood transmission interfaces, particularly veterinary procedures and husbandry training, which combine interaction duration with very close proximity, thereby increasing opportunities for exposure to respiratory droplets, aerosols, and saliva. In contrast, visitor-associated pathways generally ranked lower due to shorter interaction duration, increased separation distances, and potentially greater virus decay on fomites due to solar exposure and higher temperatures. The framework also demonstrated the value of incorporating the modifiers, interaction frequency and level of natural disinfection, which altered relative rankings even when baseline per-event likelihood was similar.

The behaviour of the framework highlights the importance of considering transmission likelihood as a multidimensional process rather than a single exposure event. These pathway rankings reflect the combined influence of exposure duration, proximity, frequency and environmental persistence captured within the framework’s assessment domains. Interaction duration and proximity strongly influenced baseline likelihood for direct pathways, reflecting established understanding of SARS-CoV-2 respiratory transmission dynamics in humans and animals [44,45]. Repeated exposure frequency further amplified transmission likelihood, particularly for routine husbandry activities, such as feeding and cleaning, that individually carried only moderate baseline likelihood. This demonstrates that cumulative low-intensity exposures may ultimately approach or exceed the relative importance of infrequent high-intensity events. Consequently, the framework may assist zoological institutions in recognising that routine operational activities, rather than only overtly high-likelihood veterinary interventions, may substantially contribute to overall transmission opportunities in captive animal environments.

Current evidence indicates that respiratory transmission through droplets and aerosols is the predominant route of SARS-CoV-2 transmission, whereas fomite-mediated transmission is considered less epidemiologically significant. Nevertheless, contaminated surfaces remain a plausible source of environmental exposure, particularly where repeated handling, prolonged viral persistence, and subsequent mucosal contact may occur.

The framework also demonstrated that environmental conditions and fomite characteristics can substantially modify relative likelihood for indirect transmission. These findings align with experimental evidence demonstrating accelerated SARS-CoV-2 decay under ultraviolet exposure and elevated temperatures [31,38,39,40]. The placement of thawed raw meat to lions is a particularly important indirect transmission interface because low temperatures substantially prolong viral persistence [36]. Similarly, fomites such as stainless steel and glass were identified as comparatively higher-likelihood materials due to their experimentally demonstrated prolonged viral survival times [31,32,33]. These findings illustrate how the framework can differentiate seasonal-, environmental-, and location-specific likelihoods, while also identifying which fomites may warrant prioritisation for cleaning, disinfection, or infrastructure modification. The framework was applied to the highest-likelihood fomite encountered within each interaction pathway rather than attempting to sum the contribution of all potential contaminated surfaces within a shared environment. While this approach represents a conservative simplification and may underestimate the cumulative contribution of multiple fomites within a single enclosure space, it provides a transparent, reproducible, and operationally practical method for identifying priority surfaces for targeted mitigation within complex zoological environments where precise quantitative estimation of indirect transmission remains challenging.

This framework is particularly relevant to emerging zoonotic pathogens for which transmission dynamics and pathogen-specific and species-specific data remain limited. Following appropriate re-parameterisation, the framework could be adapted to diverse zoological settings where enclosure design, husbandry practices, environmental conditions, and human–animal interactions may differ substantially. The highly pathogenic avian influenza represents a pertinent contemporary example, given the increasing reports of infection and mortality among mammalian species, including domestic and non-domestic felids [46]. Building on this, it could also be integrated with the hierarchy of controls [47]; identification of the factors that drive the likelihood of transmission pathways enables mitigation efforts to be directed to the points of greatest likelihood reduction, rather than prescribing an exhaustive or uniform control plan. For example, implementation of N95 respirator use would likely reduce likelihood during veterinary procedures because likelihood is driven by proximity, whereas the likelihood of brief feeding sessions is driven by frequency, which could be mitigated by shared staff rosters. This targeted approach may allow zoological institutions to allocate finite biosecurity resources more efficiently.

Several limitations of the present study should be considered in relation to the intended purpose of the framework. The framework was deliberately designed to remain practical, transparent, and adaptable for use in data-limited zoological settings. Achieving this level of usability required simplification of complex transmission processes and exclusion of variables that could not be consistently or reliably parameterised. These include host- and pathogen-related factors, such as viral load, immune status, infectious dose, and variant-specific transmissibility, as well as enclosure-specific factors, such as ventilation, airflow, and short-term microenvironmental variation [48,49,50]. Ventilation was not included as a separate scored domain because the assessed lion environments were large and naturally ventilated, and local ventilation conditions did not meaningfully distinguish the activities being compared. However, ventilation and airflow should be considered when applying the framework in smaller or more enclosed environments and when selecting mitigation measures for aerosol-associated interactions, such as respiratory protection or air filtration. Incorporating all such variables may improve biological detail but would substantially increase data requirements, complexity, and uncertainty, reducing the framework’s practical accessibility.

The indirect transmission component represents a similar pragmatic simplification. Fomite persistence was used as an indicator of relative environmental exposure opportunity, with the highest-persistence fomite encountered within each pathway selected rather than aggregating all surfaces. This approach provides a transparent and operationally practical method for comparing indirect pathways where detailed contamination and transfer data are unavailable. However, it does not model every step required for successful fomite-mediated transmission, including contamination, survival of viable virus, host contact, transfer to susceptible tissues, and infectious dose. Fomite persistence should therefore not be interpreted as equivalent to the probability of successful transmission.

As with all qualitative frameworks, pathway rankings are also influenced by interpretation of published evidence, field observations, staff-reported practices, and expert judgement [51,52]. Interaction durations and frequencies were informed by a one-day site visit and operational knowledge rather than repeated quantitative measurements, and rankings were completed by a single primary assessor.

These limitations do not detract from the framework’s principal function of supporting consistent and transparent comparison of transmission pathways so that finite mitigation resources can be directed towards the highest-priority activities. The framework is intended as a relative prioritisation approach rather than an estimate of absolute transmission probability or a formally validated risk assessment instrument.

Future studies should focus on validating and refining the framework through several complementary approaches. Structured expert assessment involving zoological veterinarians, epidemiologists, virologists, animal-care staff, and biosecurity personnel could be used to evaluate the relevance, clarity, and biological plausibility of the included domains, scoring thresholds, and modifiers. Inter-rater reliability testing across users with different professional backgrounds and levels of experience would further determine whether the framework produces consistent rankings when applied to the same transmission scenarios. Sensitivity analyses should also examine whether pathway rankings remain stable when alternative scoring thresholds, domain definitions, weighting approaches, or modifier values are applied, thereby identifying components that disproportionately influence the final ranking. Application across multiple institutions, animal species, enclosure designs, climatic conditions, and husbandry systems would allow assessment of the framework’s transferability beyond the setting examined in this study. The blank worksheet provided in the Supplementary Materials may facilitate this broader testing by enabling independent users to apply the framework consistently and document the assumptions underlying each assessment. Collectively, these approaches would provide evidence of the framework’s content validity, inter-rater reproducibility, sensitivity to parameter changes, robustness across settings, and practical utility for prioritising transmission pathways and associated mitigation measures.

## 5. Conclusions

This qualitative likelihood ranking framework provides a fit-for-purpose tool for systematically comparing SARS-CoV-2 transmission pathways between people and zoo felids, supporting consistent, defensible ranking rather than precise estimation of absolute risk. By explicitly considering duration, proximity, frequency, and environmental persistence, it highlights where likelihood is genuinely concentrated (particularly in prolonged, close-contact staff–lion interactions and low-solar-exposure environments), enabling zoos to direct biosecurity resources to the points of greatest expected impact on transmission. Because the domains are generic and adaptable, the framework may be applied to future emerging zoonotic and reverse zoonotic pathogens following parameterisation of the domains and modifiers specific to the pathogen and animal of concern, offering a pragmatic decision-support approach for data-limited settings that need rapid, transparent prioritisation of mitigation efforts across diverse zoological and One Health contexts.

## Figures and Tables

**Table 1:** Definition of transmission likelihood based on the domains of interaction duration and proximity, and SARS-CoV-2 persistence.

Domain	Definition	Likelihood Scale
**1. Interaction duration**	<30 s	Negligible
	30 s–10 min	Low
	>10–30 min	Moderate
	>30–60 min	High
	>60 min	Very High
**2a. Interaction proximity**	>10 m	Negligible
	>5–10 m	Low
	>2–5 m	Moderate
	>0.5–2 m	High
	≤0.5 m	Very High
**2b. SARS-CoV-2 persistence** **on fomite**	Not expected	Negligible
	<1 h	Low
	1–24 h	Moderate
	1–7 days	High
	>7 days	Very High

**Table 2:** Baseline propagation matrix for combining interaction duration with interaction proximity (direct transmission pathways) or SARS-CoV-2 persistence (indirect transmission pathways). The colours correspond to the level of transmission likelihood based on the five-level ordinal scale.

Interaction Duration	Interaction Proximity or SARS-CoV-2 Fomite Persistence				
	Negligible	Low	Moderate	High	Very high
Negligible	Negligible	Negligible	Negligible	Low	Low
Low	Negligible	Low	Low	Moderate	Moderate
Moderate	Negligible	Low	Moderate	High	High
High	Low	Moderate	High	High	Very high
Very high	Low	Moderate	High	Very high	Very high

**Table 5:** Parameterisation of activities across scenario tree domains and modifiers. * Transmission likelihood from human to lion only.

			Domain 1	Domain 2a	Domain 2b		Modifiers	
Person	Activity	Pathway	Interaction Duration	Proximity	Fomite with Longest Persistence	SARS-CoV-2 Survivability	Interaction Frequency	Solar Exposure & Temperature
Staff	Veterinary clinical procedures	Direct	>60 min	≤0.5 m	–	–	<Once weekly	No solar; ~20 °C
	Husbandry training	Direct	>10–30 min	0.5–2 m	–	–	Once daily	No solar; ~20 °C
	Feeding sessions	Direct	30 s–10 min	0.5–2 m	–	–	>Once daily	No solar; ~20 °C
	Moving animals between spaces	Direct	30 s–10 min	>5–10 m	–	–	>Once daily	No solar; ~20 °C
	Cleaning BOHA	Indirect	30 s–10 min	–	Stainless steel	1–7 days	>Once daily	No solar; ~20 °C
	* Feed placement	Indirect	30 s–10 min	–	Raw meat	>7 days	>Once daily	No solar; Dynamic temperature
Visitor	Viewing adjacent to mesh barrier	Direct	30 s–10 min	>5–10 m	–	–	>Once daily	Solar 9.9 MJ/m^2^/day; 20.6 °C
	* Enrichment item production	Indirect	<30 s	–	Plastic	1–7 days	<Once weekly	No solar; ~20 °C

**Table 6:** Transmission likelihood ranking of activities using likelihood propagation matrix and environmental and frequency modifiers. The colours correspond to the level of transmission likelihood based on the five-level ordinal scale * Transmission likelihood from human to lion only.

			Domain 1	Domain 2a	Domain 2b	Baseline Likelihood	Modifiers		Final Likelihood
Person	Activity	Pathway	Interaction Duration	Proximity	SARS-CoV-2 Survivability		Interaction Frequency	Solar Exposure & Temperature	
Staff	Veterinary clinical procedures	Direct	Very High	Very High	–	Very High	0	No change	**Very high**
	Husbandry training	Direct	High	High	–	High	+1	No change	**Very high**
	Feeding sessions	Direct	Low	High	–	Moderate	+2	No change	**Very high**
	Moving animals between spaces	Direct	Low	Low	–	Low	+2	No change	**High**
	Cleaning BOHA	Indirect	Low	–	High	Moderate	+2	No change	**Very high**
	* Feed placement	Indirect	Low	–	Very high	Moderate	+2	No change	**Very high**
Visitor	Viewing adjacent to mesh barrier	Direct	Low	Low	–	Low	+2	−1	**Moderate**
	* Enrichment item production	Indirect	Negligible	–	High	Low	0	No change	**Low**

**Table 7:** Environmental modifiers under each scenario.

Enclosure Space	May 2024	January/December	June
BOHA	0	−1	0
PVA	−1	−2	0

**Table 8:** Sensitivity analysis: final transmission likelihood by seasonal scenario. Environmental modifiers applied to each setting under each scenario. The colours correspond to the level of transmission likelihood based on the five-level ordinal scale.

Person	Activity	Pathway	Baseline Likelihood	Frequency Modifier	May 2024	Hottest Month (Jan)	Coolest Month (Jun)
Staff	Veterinary clinical procedures	Direct	Very high	0	**Very high**	**High**	**Very high**
	Husbandry training	Direct	High	+1	**Very high**	**High**	**Very high**
	Feeding sessions	Direct	Moderate	+2	**Very high**	**High**	**Very high**
	Shifting animals between spaces	Direct	Low	+2	**High**	**Moderate**	**High**
	Cleaning BOHA	Indirect	Moderate	+2	**Very high**	**High**	**Very high**
	Feed placement	Indirect	Moderate	+2	**Very high**	**High**	**Very high**
Visitor	Viewing adjacent to mesh barrier	Direct	Low	+2	**Moderate**	**Low**	**Moderate**
	Enrichment item production	Indirect	Low	0	**Low**	**Low**	**Low**

## Data Availability

The original contributions presented in this study are included in the article. Further inquiries can be directed to the corresponding author.

## Supplementary Materials

The following supporting information can be downloaded at: https://www.mdpi.com/article/10.3390/ani16162450/s1. Table S1—Domain category definitions. Table S2—Baseline likelihood propagation matrix. Table S3—Frequency, environmental and humidity modifiers. A blank SARS-CoV-2 transmission likelihood worksheet is provided to facilitate application of the framework.
