# Supplementary material for: A Qualitative Framework for Ranking the Relative Likelihood of SARS-CoV-2 Transmission Between People and Felids at Zoological Interfaces
Source: Animals (Basel). 2026 Aug 7;16(16):2450. doi: 10.3390/ani16162450 (PMC13508995; doi:10.3390/ani16162450)
Supplement: Supplementary file 1 [file animals-16-02450-s001.zip › animals-4379968-supplementary.pdf]

Qualitative Ranking Framework for Human-Felid SARS-CoV-2 Transmission in Zoological Settings

This worksheet is based on the scenario-tree (Figure 1) and propagation-matrix framework described by Tang et al. (2026). Complete one row for each activity or transmission pathway assessed. Mitigation measures, including personal protective equipment and physical barriers, should be excluded at this stage so that the inherent transmission likelihood can be ranked and activities requiring prioritised mitigation can be identified.

How to complete this worksheet

Step 1. Classify the transmission pathway - Determine whether the activity represents a direct or indirect interaction.

Step 2. Score Domain 1: Interaction duration - Use Table S1 to assign an interaction-duration category ranging from Negligible to Very High.

Step 3. Score Domain 2a or Domain 2b - For direct pathways, score interaction proximity using Table S1. For indirect pathways, score SARS-CoV-2 persistence on the highest-likelihood fomite involved using Table S1.

Step 4. Determine the baseline likelihood - Use Table S2, the propagation matrix, to cross-reference the relevant domain categories:

- For direct pathways, cross-reference Domain 1 and Domain 2a.
- For indirect pathways, cross-reference Domain 1 and Domain 2b.

The resulting category represents the baseline likelihood.

Step 5. Score the modifiers - Use Table S3 to score:

- Interaction frequency over a one-week assessment period;
- Solar exposure and temperature; and
- Relative humidity

Step 6. Apply the modifiers to the baseline likelihood - Upward and downward modifiers offset one another. Calculate the net number of levels and adjust the baseline likelihood accordingly along the ordinal scale: Negligible < Low < Moderate < High < Very High. The final likelihood cannot be adjusted below Negligible or above Very High.

Step 7. Record the final likelihood - Record the final likelihood category for the activity and repeat the process for each activity or pathway within the setting being assessed.

Table S1 – Domain category definitions

Domain	Negligible	Low	Moderate	High	Very High
**1. Interaction duration**	< 30s	30s– 10 min	>10–30 mins	> 30–60 mins	> 60 mins
**2a. Interaction proximity (direct pathways)**	>10 m	> 5–10 m	> 2–5 m	> 0.5–2 m	≤ 0.5 m
**2b. SARS-CoV-2 persistence on fomite (indirect pathways)**	Not expected	< 1 hour	1–24 hours	1–7 days	> 7 days

Table S2 – Baseline likelihood propagation matrix

		**Interaction proximity (Domain 2a) or SARS-CoV-2 fomite persistence (Domain 2b)**				
		**Negligible**	**Low**	**Moderate**	**High**	**Very High**
**Interaction duration (Domain 1)**	**Negligible**	Negligible	Negligible	Negligible	Low	Low
	**Low**	Negligible	Low	Low	Moderate	Moderate
	**Moderate**	Negligible	Low	Moderate	High	High
	**High**	Low	Moderate	High	High	Very High
	**Very High**	Low	Moderate	High	Very High	Very High

Table S3 – Frequency, environmental and humidity modifiers

Modifier	Condition	Modifier
**Frequency of interaction (over 1-week period)**	< Once weekly	No change (0)
	Between once weekly and once daily	Increase 1 level (+1)
	> Once daily	Increase 2 levels (+2)
**Solar exposure & Temperature**	Strongly reducing: high solar exposure ≥22 MJ/m^2/day and/or mean temp ≥28°C	Reduce 2 levels (-2)
	Reducing: any natural solar exposure <22 MJ/m^2/day and/or mean temp >23–<28°C	Reduce 1 level (-1)
	Neutral: no solar exposure and mean temp 15–23°C	No change (0)
	Enhancing: cool mean temp 10–<15°C	Increase 1 level (+1)
	Strongly enhancing: cold <10°C, refrigerated or frozen storage	Increase 2 levels (+2)
**Relative humidity (RH)**	RH 40–80%	No change (0)
	RH <40% or >80%, judged persistence-enhancing	Increase 1 level (+1)

Worksheet – Qualitatively rank transmission likelihood of interactions

Facility and enclosure: ____________________ Species: ____________________

Assessment start date____________________ Assessor: ____________________

Assessment end date____________________

List every distinct human–felid activity/pathway at your facility (e.g. veterinary procedures, feeding, husbandry training, cleaning, moving animals, public viewing). Use Tables S1–S3 above to complete each row.

**Activity / pathway**	**Direct/ Indirect**	**Duration category (Table S1)**	**Proximity or fomite persistence category (Table S1)**	**BASELINE likelihood (Table S2)**	**Frequency modifier (Table S3)**	**Solar exposure & Temperature (Table S3)**	**Relative humidity (Table S3)**	**OVERALL modifier**	**FINAL transmission likelihood**
**1.**									
**2.**									
**3.**									
**4.**									
**5.**									
**6.**									
**7.**									
**8.**									
**9.**									
